# WWW design code – a new tool for colour estimation in animal studies

**DOI:** 10.1186/1742-9994-1-2

**Published:** 2004-10-06

**Authors:** Åsa Berggren, Juha Merilä

**Affiliations:** 1 Ecology Group, Massey University, Private Bag 11 222, Palmerston North, New Zealand; 2 Department of Biological and Environmental Sciences, P.O. Box 65, FI-00014 University of Helsinki, Finland; 3 Present Address: Department of Entomology, P.O. Box 7044, Swedish University of Agricultural Sciences, SE-75007 Uppsala, Sweden

## Abstract

**Background:**

The colour of animals' skin, fur, feathers or cuticula has been estimated in a large number of studies. The methods used to do so are diverse, with some being costly and not available to all researchers. In a study to measure plumage colour in a bird species, a new method of creating a colour chart was developed. While colour-charts have their own limitations, these can be minimised when they have the following properties: 1) being readily available to the majority of biologists, 2) containing a large array of colours to allow accurate recording and differentiation of subtle colour differences, 3) low cost, 4) adhering to a world-wide standard, and 5) being available in both hard-copy and digital formats to allow for various analytical methods. The method described below satisfies all of these requirements.

**Results:**

Colour charts estimated to fit the range of the species' plumage colours were created on the computer screen using web software that allowed for HTML-coding (in this case Dreamweaver™). The charts were adjusted using feathers from dead specimens until a satisfying range of darker and lighter colours were found. The resulting chart was printed out and was successfully used in the field to determine the plumage colour of hand-held birds.

**Conclusion:**

Access to a computer and printer, and the software to enable the creation of a chart, is within the reach of the vast majority of biologists. The numbers of colours that can be generated should suit most studies, with the advantage of the method being that the chart can be individually tailored to the species under study. HTML colour coding is a worldwide standard, thus the colours used in studies can be described in the methods section of journal articles using the six-digit alphanumeric code. We believe this method is very useful as a low-tech method for future estimation of individual colour.

## Background

Animal studies from diverse fields – such as morphology, physiology, behaviour, population dynamics, genetics, ageing and sexing – often require the estimation or classification of colour in skin, fur, feathers or cuticula. To make meaningful comparisons within and between studies, and effectively communicate these findings, a standardised method of assigning a colouration is required. Colour can be categorised, scored and ranked without recourse of any aid other than the observer's eye, as has been successfully demonstrated in many studies [1-4]. Aids to indexing and labelling colours have traditionally compared animals to published colour-standard cards, charts or books. These were often generated for other purposes, such as paint making or characterising soil types [5-8]. Such charts have been employed since the early 1800's, with Charles Darwin using Werner's colour charts [9] on the *Beagle *expedition [10]. Newer electronic measuring tools, using a reflectance spectrophotometer to give a high precision estimate of hue, saturation and brightness of the colour [11-14], have been recommended as a reliable and objective way of acquiring detailed data on different aspects of colour [15]. Lately, photo-processing software (e.g. Adobe Photoshop^® ^– Adobe Systems Inc., San Jose, CA, USA) has been used in estimating colour brightness, saturation and hue from digitised photos [11,16].

The benefit of using highly technical methods is the ability to gain very detailed and precise information on different aspects of colour that may be impossible to detect using the human eye. However, in many studies such a high level of precision is not required, and the equipment may be impractical in the field or beyond the budget of the research group. Thus despite the option of high-tech measurement methods, visual comparisons to standardised colour charts are still practical and valuable for many field biologists. While colour-charts have their own limitations, these are minimised when they have the following properties: 1) availability to the majority of biologists, 2) large array of colours to allow accurate recording and differentiation of subtle colour differences, 3) low cost, 4) adherence to a world-wide standard, and 5) availability in both hard-copy and digital formats to allow for various analytical methods. Below we describe a method that satisfies all of these requirements.

## Results and Discussion

**Figure 1:**
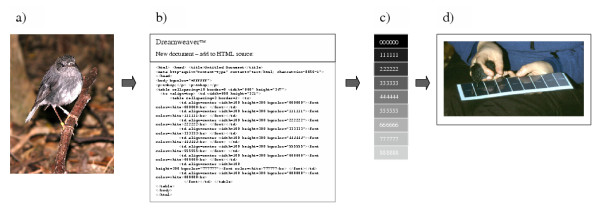
The making of a HTML coded colour chart for estimation of animal colour in the field. First the colours relevant for the study are estimated from samples from the species of interest (in this case the New Zealand robin (*Petroica longipes*)) (a). From the sample, a set of colours is coded using HTML coding (b), which has a specific code for every colour. In this case the software Dreamweaver™ was used to create nine different shades ranging from light grey (888888) to black (000000) (c). When the chart is made it is printed out and can be enclosed within a plastic covering for protection. This procedure worked well for colour estimation of the species in focus, with individuals' colours being easy to index under field conditions (d).

A starting point for development of this new method was a study on plumage colour in an endemic New Zealand passerine – North Island robin (*Petroica longipes*) – where there was a need for a reliable, species-specific and cost-efficient method to estimate colour (Fig. 1; Å Berggren unpublished data). The birds were to be caught and handled in the field, but could not be moved and no samples from the plumage were to be taken. Hence, it was decided to create a specific colour chart, unique for the colours to be compared in this study, and easy to use under fieldwork conditions. It was decided that a computer-generated colour-chart would ideally suit the purposes of the study.

The light emitted from a computer monitor is composed of a particular combination of red, green, and blue light. The proportion of each of these components in the visual colour spectrum can be expressed as a number unique for each specific colour or hue. These colours are coded for using the HTML (Hypertext Markup Language) computer language. This system of colour coding was developed for the Netscape^® ^web-browser (Netscape Communication Corp.) and has since become the industry standard [17]. Today, colour coding is one of the most important features in web page design when creating informative and graphically appealing sites [18]. Each HTML colour-code specifies the composite of a colour with a six-digit alphanumeric code where the first two digits represent the amount of red, the middle two the amount of green, and the last two the amount of blue. Each character may be represented in one of 16 ways (0 – 9 and A – F), creating a vast array of potential colours [17]. The actual number of colours that can be produced for viewing on the screen is limited by the computer, ranging from 256 colours in older computers to 16.7 million in newer models. These colours can be generated in web browser software such as Netscape^®^, by using the composer feature, or through web-design programmes (e.g. Dreamweaver™ – Macromedia Inc. or FrontPage^© ^– Microsoft Corp.). The colours can be displayed on the computer screen and fine-tuned by adjusting the HTML code. For example, between the colours "0000FF" for blue, "00008B" for dark blue and "00C78C" for turquoise blue, a large number of other blues can be created and viewed. When a suitable set of colours have been decided upon, they can be printed out as they present themselves on the screen and saved for future use.

The focus species of the plumage study, the North Island robin, has a brown-grey-black plumage (Fig. 1) [19]. Using HTML coding, a colour chart was developed to match the natural variance in the species' brown to black plumage. To ensure the colour range of the feathers was accurately represented in the chart, feathers from a dead specimen were used and compared to the computer generated colours. From this point, an equal number of brighter and darker colour gradations were created, centring on the colour of the feather specimens. The HTML-coding numbering from 0 to 8 (000000 – 888888) resulted in nine shades from black to light grey (Fig. 1). This allowed a progression in equal steps from lighter to darker to be displayed sequentially on a printout. This made it possible to hold the bird next to the chart and move it until a colour match was made (Fig. 1). The technique worked well and it was possible to get an accurate colour ranking of the darkness of the plumage for the captured 32 birds (Å Berggren unpublished).

## Conclusions

The method of colour-chart creation utilising HTML code satisfies the criteria listed above. Access to a computer and printer, and the software to enable the creation of a chart, is within the reach of the vast majority of biologists. The numbers of colours (and patterns) that can be generated should suit most studies, with the advantage of the method being that the chart can be individually tailored to the species under study. HTML colour coding is a worldwide standard, thus the colours used in studies can be described in the methods section of journal articles using the six-digit hexadecimal code. Comparisons are not limited to a printout of a colour chart, digital images can also be compared and their colours scored using this method. Drawbacks may include identifying the right colours for the chart to accurately match the animals as encountered in the field, a problem equivalent to other printed colour charts. It is also possible that when printing, the printed colours differ from the colour range as displayed on the computer screen. With some people still using older computers, which are not able to display colours coded in newer machines, there is a risk that there is a discrepancy between computers in the colour presented on the screen. This may be a problem when the aim is to compare specific colours between different studies, but not an issue within studies. Fortunately, this problem will decrease with more computer system being able to display the full range of 16.7 million colours. When using the colour-charts in the field, the usual care of indexing individuals under the same lighting conditions should be taken [15]. As the technique is easy to refine and adjust to the requirements needed for the species, we believe it is very useful as a low-tech method for future estimation of individual colour. We encourage other researchers and field workers to try the method in future colour studies.

## Authors' contributions

JM came up with the initial idea of using web designer tools for creating colour charts. ÅB developed the colour charts using HTML coding and used them in the field as a research tool. ÅB and JM wrote the manuscript, with ÅB doing the major part. All authors read and approved the manuscript.
